# Reducing Prenatal Alcohol Exposure and the Incidence of FASD: Is the Past Prologue?

**DOI:** 10.35946/arcr.v43.1.02

**Published:** 2023-04-20

**Authors:** Grace Chang

**Affiliations:** Department of Mental Health, Veterans Administration Boston Healthcare System, Boston, Massachusetts, Department of Psychiatry, Harvard Medical School, Boston, Massachusetts

**Keywords:** alcohol, prevention, fetal alcohol effects, fetal alcohol syndromes, preconception care, fetal alcohol spectrum disorders

## Abstract

**PURPOSE:**

This narrative review summarizes and synthesizes the clinical trials and randomized clinical trials that evaluated selected and targeted approaches to reducing preconception and prenatal alcohol exposure (PAE) and alcohol-exposed pregnancy (AEP) since 2011.

**SEARCH METHODS:**

A professional hospital librarian completed the primary search using strategies specified within this review, resulting in 94 records returned in PubMed, Ovid MEDLINE, Clinical Key, the World Health Organization International Clinical Trials Registry Platform, and ClinicalTrials.gov. The author completed two supplementary literature searches.

**SEARCH RESULTS:**

From the total of 238 records returned from the three searches, 217 records were eliminated. Elimination reasons included other medical problem (119); duplicate entry (34); no content/results (23); secondary analysis (16); focus on effects of PAE (9); treatment of childhood fetal alcohol spectrum disorders (FASD) (6); maternal risk factors (3); and other (7). The remaining 21 studies were included with four overarching themes: (1) case management efforts (*n* = 4); (2) preconception efforts to reduce AEP (*n* = 5); (3) motivational interviewing and screening, brief intervention, and referral to treatment (*n* = 2); and (4) use of technology to deliver the intervention (*n* = 10).

**DISCUSSION AND CONCLUSIONS:**

Case management and home visits did not appear to have strong current empirical support. Study limitations included small sample sizes and no comparison groups, whereas larger efforts did not demonstrate definitive advantages to justify this intensive approach. The studies of preconception efforts, all based on the Project CHOICES approach, had similar outcomes, with the reduction in AEP risk largely due to improved contraception in women of childbearing age who were sexually active and drank alcohol but were not pregnant. It is unknown whether these women refrained from alcohol use when they became pregnant. Two studies of motivational interviewing to reduce prenatal alcohol use did not demonstrate the efficacy of the intervention. Both were small, with less than 200 pregnant women combined; moreover, the study samples had low baseline levels of alcohol use, allowing little opportunity for improvement. Finally, studies evaluating the impact of technological approaches to reducing AEP were reviewed. These exploratory investigations had small sample sizes and provided preliminary evaluations of techniques such as text messages, telephone contact, computer-based screening, and motivational interviewing. The potentially promising findings may inform future research and clinical efforts. Future directions may include research to address the limitations of the evidence to date and should reflect the complexities of FASD that include the biological and social context associated with prenatal alcohol use.

Prenatal alcohol exposure (PAE) is linked to miscarriage, stillbirth, preterm birth, sudden infant death syndrome, and fetal alcohol spectrum disorders (FASD).^1^ Although PAE is the sole necessary cause of FASD, the etiology of this leading preventable cause of disability is multifaceted and complex, including lifestyle, maternal, sociodemographic, social, gestational, and genetic risk factors.^2^–^4^ As the identification of specific maternal drinking behaviors related to FASD remains inconclusive, efforts to reduce PAE and the incidence of FASD continue to be necessary.^5^,^6^

The last issue of *Alcohol Research: Current Reviews* dedicated to FASD was published in 2011, when the journal was named *Alcohol Research & Health*. At that time, maternal risk factors for FASD were recognized to be multidimensional and included quantity, frequency, and timing of alcohol exposure; maternal age; and social relationships, among others. Because it was not known then which factors were most likely to lead to having children with FASD,^7^ prevention efforts were important. Universal approaches—such as broad media campaigns and warning labels on alcohol beverage containers—were not particularly effective in reducing alcohol use during pregnancy. Limited research was available on selected and targeted prevention efforts aimed at women in special risk groups or at women known to be more vulnerable because of binge drinking. However, screening instruments to identify women at risk of prenatal alcohol use and administration of brief interventions in the clinic had positive effects in reducing drinking during pregnancy.^8^

In 2014, the World Health Organization (WHO) published *Guidelines for the Identification and Management of Substance Use and Substance Use Disorders in Pregnancy*.^9^ The Guidelines were developed to enable professionals to assist women who were pregnant and who used alcohol or other drugs to optimize healthy outcomes for their patients and the fetus or infant. The Guidelines reflected the collaboration of the WHO internal steering group, Guideline Development Group, and External Review Group. Based on the Grading of Recommendations Assessment, Development, and Evaluation (GRADE) system for assessing quality of evidence, the Guidelines focused on six areas, two of which are relevant to this review: (1) screening and brief intervention (SBI), and (2) psychosocial interventions to prevent PAE and the incidence of FASD.

Because much of the evidence supporting the effectiveness of SBI predated the GRADE standards, the WHO Guidelines had “strong” recommendations for SBI despite “low” quality of evidence. SBI was supported because the potential benefits outweighed the potential harms. On the other hand, psychosocial interventions for substance use disorders in pregnancy received only a “conditional” recommendation because of “very low” evidence to support their use. Examples of psychosocial interventions include motivational interviewing and home visits following delivery to support abstinence.

The WHO recommendations are reflected in the 2015 *Committee Opinion* of the American College of Obstetricians and Gynecologists,^10^ which provided an ethical framework to encourage physicians to use routine screening, brief intervention, and referral to treatment (SBIRT) for alcohol and other substance use among their obstetrics/gynecology patients as appropriate. Similarly, the Society of Obstetricians and Gynaecologists of Canada published a clinical practice guideline in 2020, which recommended that all pregnant women be asked about alcohol consumption using evidence-based SBI approaches.^11^

While the most prudent recommendation continues to be abstinence from alcohol throughout pregnancy, some pregnant women continue to drink alcohol.^12^,^13^ Past 30-day reports of alcohol use by pregnant women between 2018 and 2020 indicated that 14% reported current drinking (i.e., at least one drink in the past 30 days) and 5% reported binge drinking (i.e., four or more drinks in one sitting, at least once in the past 30 days).^14^ Both measures were 2 percentage points higher than the 2015–2017 rates, but these changes were not statistically significant.^14^

Moreover, some research has suggested that reports about past 30-day alcohol use by pregnant women underestimate the true extent of prenatal alcohol use. Among 4,088 randomly selected control mothers in the National Birth Defects Prevention Study, 30% had “some alcohol” while pregnant and 8% reported binge drinking (defined as four or more alcoholic drinks per occasion).^15^ The Generation R Study, a population-based prospective cohort study from fetal life to adulthood of 7,141 individuals in the Netherlands, found that 37% of pregnant women continued to consume alcohol after pregnancy was known.^16^

These rates of alcohol use during pregnancy stand in contrast to the Healthy People 2030 goals established by the U.S. Department of Health and Human Services. Healthy People 2030 sets data-driven national objectives to improve health and well-being over the next decade, including the goal of increasing abstinence from alcohol among pregnant women to 92%.^2^

This narrative review focuses on the clinical trials and randomized clinical trials that have evaluated selected and targeted approaches to reducing preconception alcohol exposure, PAE, and AEP since 2011, when *Alcohol Research: Current Reviews* (then called *Alcohol Research & Health*) last focused on this area. Examples of these approaches include case management for pregnant women at risk of drinking; applications of the Project CHOICES preconception approach to reduce AEP by adoption of effective contraception in clinical and other settings; motivational interviewing among pregnant women; and utilization of technology-based interventions such as the telephone, Internet, and text messaging to reduce prenatal alcohol exposure. The focus on clinical and psychosocial efforts was chosen because these approaches are still needed to reduce AEP and the prevalence of FASD, to establish a new baseline for inquiry, and to encourage innovations moving forward.

## Search Methods

A literature search was conducted in November 2021 of the PubMed, Ovid MEDLINE, Clinical Key, WHO International Clinical Trials Registry Platform (ICTRP), and ClinicalTrials.gov databases using these search strategies:

- For PubMed: (“alcohol drinking”[MeSH Major Topic] OR prenatal alcohol[Title/Abstract]) AND (“Pregnancy”[MeSH Terms] OR “Prenatal Care”[MeSH Terms]) AND (“clinical trial”[Publication Type] OR “clinical trial, phase I”[Publication Type] OR “clinical trial, phase II”[Publication Type] OR “clinical trial, phase III”[Publication Type] OR “clinical trial, phase IV”[Publication Type] OR “controlled clinical trial”[Publication Type] OR “pragmatic clinical trial”[Publication Type] OR “randomized controlled trial”[Publication Type]);
- For Ovid MEDLINE: (prenatal* adj5 (alcohol or ethanol)). tw. limited to (clinical trial, phase I or clinical trial, phase II or clinical trial, phase III or clinical trial, phase IV or clinical trial or controlled clinical trial or pragmatic clinical trial or randomized controlled trial);
- For Clinical Key: “prenatal alcohol” filtered by Clinical Trials;
- For WHO ICTRP and ClinicalTrials.gov: “prenatal alcohol.”

The search was limited to articles published in English over the last 10 years. A professional librarian (see Acknowledgments) at the author’s hospital executed these foundational literature searches resulting in 94 hits.

In addition, the author completed two supplementary literature searches using PubMed and the search terms—(1) prevention and fetal alcohol spectrum disorders (FASD), and (2) PCAP (parent child assistance program), which resulted in 19 and 125 hits, respectively. These additional literature searches were undertaken to include prevention of FASD and the parent-child assistance program studies that were not returned by the other searches but are a case management approach in the United States well described in the decade before 2011.^17^

Table 1 summarizes the literature tracking.^18^,^19^ Of the total of 238 records returned by the three searches, 217 records were eliminated. Elimination reasons included: other medical problem (119), duplicate entry (34), no content/results (23), secondary analysis to allow consistent focus on primary investigations (16), focus on PAE effects (9), treatment of childhood FASD (6), maternal risk factors (3), and other (7). A total of 21 studies were thus included.

## Results

Table 2 summarizes the 21 studies selected for review. They are sorted into four overarching themes: (1) case management efforts, (2) preconception trials to reduce AEP, (3) motivational interviewing and SBIRT, and (4) use of technology.

### Effectiveness of Case Management Efforts

Several clinical trials evaluated case management or home visits as an intervention to reduce prenatal alcohol use. Most relied on motivational interviewing and community reinforcement. Two were unblinded, indicated prevention efforts without comparison groups,^20^,^21^ whereas the other two used a randomized controlled trial design.^22^,^23^

May et al. reported on a sample of 41 women from the Cape provinces of South Africa who were deemed to be at high risk for bearing a child with FASD; 88% of the women were pregnant at intake.^20^ Women were considered at high risk if they (1) had already borne one child with FASD or had drank heavily during a prior pregnancy; (2) were currently drinking eight or more drinks per week or one binge of three or more drinks per day; or (3) had a “high score” on the self-administered questionnaire (score > 2) or the Alcohol Use Disorders Identification Test (AUDIT) (score > 8). They were offered 18 months of case management (CM) with data collected at baseline and at 6, 12, and 18 months after starting CM; there was 27% loss to follow-up before 18 months. The AUDIT was used to measure drinking during the study. For the 29 women involved in CM for the entire 18 months of the study, the mean AUDIT score was 19.4 (*SD* = 6.7) at baseline, dropped to 9.7 at 6 months, rose to 10.8 at 12 months, and 12.3 at 18 months. Limitations include the indirect measure of alcohol use, high attrition rate, lack of a comparison group, and small sample size. It also was unclear whether the case management strategies are feasible with regards to resources in other settings (e.g., clinical rather than research).

De Vries et al. conducted a similar prospective intervention study at community health clinics in the Western Cape province of South Africa between January 2009 and June 2011.^21^ The researchers offered CM to support pregnant women who drink heavily to achieve abstinence or reduction in alcohol during pregnancy and in the first postpartum year. These investigators used the same definition of “heavy drinking” and risk of having a child with FASD.^20^ CM incorporated life management, motivational interviewing techniques, and the community reinforcement approach. A total of 67 women enrolled, most during their second trimester and all at high risk for bearing a child with FASD; loss to follow-up at 18 months was 24% loss. The researchers found that compared with baseline, alcohol use decreased from the time of enrollment to the first 6 months of CM, but increased at 12 and 18 months. Limitations were similar to those mentioned for the study by May et al.^20^

Rotheram-Borus et al. examined the effect of a one-time home visiting intervention on prenatal alcohol use and problematic drinking, as well as the association of home visiting and alcohol use on children’s behavioral, cognitive, and health outcomes among 1,236 mothers and their children from pregnancy to 5 years afterwards.^22^ The study, which was conducted in Cape Town, South Africa, included 1,236 mothers and their children who participated in a program that allowed up to 5 years of contact. The study compared standard care and the intervention, known as the Philani Program, using a longitudinal cluster-randomized design. The Philani Program trained township women as Mentor Mothers who offered a brief, one-visit intervention on alcohol prevention in pregnancy, in addition to their other home-based primary-care efforts, such as rehabilitation of underweight children or prevention of mother-to-child HIV transmission. At baseline, 10% of the pregnant women in both conditions drank alcohol, and both groups also showed reductions in alcohol use over the course of pregnancy and increases in alcohol use afterwards. There were no statistically significant differences in alcohol use between the groups at any time with this brief, one-time intervention.

More recently, Catherine et al. reported findings from the Nurse-Family Partnership (NFP), a program in which public health nurses provided frequent home visits from early pregnancy until the children were 2 years old.^23^ With a focus on first-time parents experiencing socioeconomic disadvantage, the analysis included 739 pregnant girls and women ages 14 to 24 who were randomly allocated 1:1 to the intervention (NFP plus treatment as usual) or control (treatment as usual). NFP guidelines allowed as many as 14 prenatal visits and 50 postpartum visits, during which nurses helped participants to identify and meet health and social goals, including reducing prenatal substance use. Changes in the use of nicotine cigarettes and alcohol use by 34–36 weeks’ gestation were prespecified prenatal secondary outcomes. The research team found no evidence that NFP was effective in reducing rates of prenatal use of cigarettes or alcohol; the intervention condition was associated with reduced cannabis use.

In conclusion, the few published studies of CM approaches to reducing prenatal alcohol use did not provide strong empirical support for the effectiveness of these measures. These findings are consistent with the assessment published in the WHO Guidelines.^9^

### Effectiveness of Preconception Measures to Prevent AEP

Four randomized trials and one clinical trial of efforts to reduce AEP, some of which were based on the CHOICES approach,^24^ were published in the past decade. These efforts used motivational interviewing and cognitive behavior strategies and targeted the adoption of effective contraception and reduction of alcohol use.

Rendall-Mkosi et al. randomized 165 nonpregnant women who were considered to be at risk for AEP to either a five-session motivational interviewing intervention or a control condition.^25^ Women were classified as being at risk if they (1) were ages 18 to 44, (2) were not pregnant, (3) had engaged in risky drinking in the past 3 months (defined as more than five drinks per episode or more than seven drinks per week), (4) had used ineffective or no contraception, (5) were able to conceive, (6) had vaginal sex in the past 3 months, and (7) lived within a certain distance from the main town. The study originally included a third arm with a group-based life-skills training intervention, which was terminated because of difficulties with implementation. Although modeled on Project CHOICES, this study used simplified data collection tools at baseline and at 3 and 12 months afterward. The main finding was that women in the motivational interviewing group were more than twice as likely as women in the control group to lower their risk for AEP at 12-month follow-up (*OR* = 2.64, 95% *CI* [1.18, 5.94]); this difference was reduced but remained significant using an intention-to-treat analysis (*OR* = 2.19, 95% *CI* [1.05, 4.65]). However, the reduction in risk for AEP was due mainly to the improved use of contraceptives rather than a reduction in alcohol use.

Ingersoll et al. tested a one-session motivational AEP prevention intervention among 217 women who had at least one episode of unprotected vaginal sex with a male partner and drank at risky levels (defined as more than three drinks per occasion or more than seven drinks per week) in the past 90 days.^26^ The women completed baseline assessments and were randomized to motivational interviewing plus assessment feedback (EARLY), informational video, or informational brochure conditions. Outcomes were drinks per drinking day, ineffective contraception rate, and AEP risk at 3 and 6 months. Results showed reductions in drinking, increased contraception, and reduced AEP risk for all conditions. Because all conditions included an assessment of baseline drinking behaviors, it appears that raising risk awareness through assessment could be impactful. The effect from assessment on subsequent alcohol use is supported by other studies.^27^

Sobell et al. conducted a CHOICES-like randomized controlled study for 354 women at risk for AEP; participants included 145 college students.^28^ Risk for AEP was defined as being of childbearing age (18–45 years old), having had heterosexual vaginal intercourse with ineffective contraception, and having consumed eight or more standard drinks per week or five or more standard drinks per episode. (Unless otherwise indicated, a standard drink is defined as containing 14 g of alcohol and corresponds to 12 oz of beer [5% alcohol], 5 oz of wine [12% alcohol], or 1.5 oz of liquor or spirits [40% alcohol].) The women were randomized either to receive motivational feedback based on Project CHOICES or to a control group receiving information only. Similar to the findings by Ingersoll et al.,^26^ there was no significant difference between the two interventions at 6-month follow-up. For all groups, risk reduction occurred primarily through increasing effective contraception.

Velasquez et al. tested the efficacy of CHOICES Plus, a preconception intervention for reducing the risk of AEP and tobacco-exposed pregnancy, among 261 nonpregnant women of childbearing age (18 to 44 years) attending primary care clinics in a large Texas public health system.^29^ The women were sexually active, with at least one episode of unprotected vaginal sex with a male partner, and were considered to show risky drinking (defined as more than three drinks per episode or more than seven drinks per week). In this study, women were randomized to either two CHOICES Plus sessions and a contraceptive visit or to brief advice and referral to community resources. In an intention-to-treat analysis at 9-month follow-up, the Project CHOICES Plus group was more likely than the brief-advice group to reduce the risk of AEP, with an incidence rate of 0.620 (95% *CI* [0.511, 0.757]) and an absolute risk reduction of −0.233 (95% *CI* [−0.239, −0.226]). The Project CHOICES group at risk for both exposures was also more likely to reduce risk of tobacco-exposed pregnancy (incidence rate ratio, 0.597; 95% *CI* [0.424, 0.840]), and absolute risk reduction of −0.233 (95% *CI* [−0.019, −0.521]).

Hanson et al. tailored the Project CHOICES approach for a prevention program run by the Oglala Sioux Tribe so that the program was culturally appropriate for American Indian women.^30^ The program’s effectiveness was examined in two communities on the reservation and one community off the reservation. A total of 193 nonpregnant Native American women were enrolled, and 51% completed baseline assessment and both 3- and 6-month follow-up; there was no comparison group. The participants were considered at risk for AEP because they exceeded low-risk drinking limits for women (defined as four or more drinks per occasion or eight or more drinks per week) and were not using effective contraception while sexually active. Results were consistent with the findings of other CHOICES-related research; thus, women receiving the Oglala Sioux Tribe CHOICES intervention were significantly more likely to improve birth control (68% at 3 months and 62% at 6 months) than to drink less (10% and 20% reductions in binge drinking at 3 and 6 months, respectively).

In conclusion, the preconception Project CHOICES approach to preventing AEP appears to exert its effect primarily through improving effective contraception in women of childbearing age who are sexually active, rather than through reducing alcohol use. Strengths of the studies assessing the CHOICES approach include the use of consistent approaches to the intervention and, in most cases, use of comparison groups.

### Effectiveness of Motivational Interviewing and SBIRT

In the past decade, two studies assessed the effectiveness of motivational enhancement approaches based on screening and brief intervention in decreasing prenatal alcohol use.

Osterman et al. examined the usefulness of a single-session motivational interviewing intervention in decreasing alcohol use during pregnancy.^31^ The intervention employed theory-based mechanisms of behavior change as guided by the self-determination theory. The study included 67 pregnant women who reported past year alcohol use, 59 of whom were randomized either to the intervention or to a comparison group; all participants completed baseline and follow-up interviews. The intervention was not found to be effective in decreasing prenatal drinking behaviors. In a second study, the same research team tested the effectiveness of a single-session motivational interview to decrease alcohol use during pregnancy.^32^ This study included 122 pregnant women who drank in the past year who were randomized to either the intervention or a comparison group. Treatment effects over time were evaluated with Poisson and linear regression with generalized estimating equation. Again, motivational interviewing was not found effective in decreasing prenatal alcohol use. The investigators suggested that the low levels of baseline alcohol use in the study sample left little room for improvement. A secondary analysis by the same research group is beyond the scope of this review.^33^

These two studies, both of which were limited by small participant numbers, indicate that the promise of motivational interviewing or screening and brief intervention in reducing prenatal alcohol use cannot be confirmed. The two trials do not demonstrate that a single-session intervention will influence behavior over the course of a pregnancy.

### Effectiveness of Technology-Based Interventions

Several trials evaluated the impact of text messages, brief intervention delivered over the telephone, computer-delivered screening and brief intervention, and an Internet intervention delivered preconception on risk of AEP. Additionally, one study assessed a novel application of four-dimensional (4D) ultrasound of fetal development as an intervention.

#### Text messages

Evans et al. conducted two pilot studies of the mobile health program Text4baby in two groups of women. One group included 123 women with low income seeking prenatal care;^34^ the other included 943 pregnant military women presenting for care at a military medical center.^35^ Text4baby is a mobile health program based on social cognitive theory in which health messages are delivered to pregnant women and new mothers to improve their health care beliefs and behaviors with the goal of improving health status and clinical outcomes. In both studies, the women completed a baseline assessment survey before being randomized to the intervention group (Text4baby plus usual care or usual care alone). Follow-up was planned at approximately 28 weeks’ gestation for the low-income group and 4 weeks after enrollment for the military group.

In the study of women with low income, 73% participated at follow-up. The Text4baby intervention was significantly associated with increased agreement with the statement, “I am prepared to be a new mother,” between baseline and follow-up (*OR* = 2.73, 95% *CI* [1.04, 7.18], *p* = .042). Furthermore, among mothers of low income with a high school education or higher, the intervention was significantly associated with increased agreement with attitudes against alcohol consumption during pregnancy (*OR* = 2.80, 95% *CI* [1.13, 6.90], *p* = .026).^34^

The study involving military women had greater loss to follow-up, with only 49% of participants completing the assessment at 4-week follow-up. Moreover, the results were less encouraging. In a generalized estimating equations logistic regression model adjusted for four socioeconomic variables, imputations for missing values for marital status and race, and inverse probability weighting to account for attrition, there were marginally significant effects for improved strong agreement with the statements, “If I visit my health care provider on a regular basis, I will be a healthy new mother” as well as “Drinking alcohol will harm the health of my developing baby.” The Text4baby intervention had no effects on any of the measured behaviors (e.g., alcohol use).

#### Telephone-based interventions

Two studies evaluated a telephone-based preconception intervention for women of childbearing age who were sexually active, did not use effective contraception, and consumed alcohol at “risky levels.” Farrell-Carnahan et al. offered a one-session, remote-delivered, preconception, motivational interviewing-based AEP intervention (EARLY Remote) to 46 non–treatment-seeking community women;^36^ there was no comparison group. The participants were women who were sexually active and consumed seven or more standard drinks per week and/or three or more standard drinks per drinking episode in the past 90 days and who did not use reliable contraception. All participants received the baseline, 3-month, and 6-month assessments via telephone. Both the number of drinks per drinking day and the rate of unreliable contraception decreased over time.

The study by Wilton et al. included 132 women ages 18 to 44 who screened positive for drinking at risky levels (defined as more than seven drinks per week or more than three drinks in any one day) and who were not using effective contraception.^37^ After completing a baseline assessment interview, the women were randomized to a brief two-session intervention delivered either via telephone or in person. There was no significant difference between the two groups in the success of the brief intervention at 6-month follow-up. Overall, participants demonstrated small reductions in alcohol use (11%) and larger increases in the effective use of contraceptives. The intervention modality was not a significant predictor of any outcomes after controlling for potential confounding measures.

#### Computer-based interventions

Several computer-based interventions have been evaluated in preconception and pregnant women. Van der Wulp et al. completed a cluster randomized trial in which 60 Dutch midwifery practices were randomly assigned to one of three conditions: health counseling, computer-tailored feedback, and usual care.^38^ Participating women needed to understand Dutch, be age 18 or older, be no more than 12 weeks pregnant, and have consumed alcohol while pregnant. Among the participants, 135 women received counseling from their nurse-midwife according to a health-counseling protocol that included seven steps in three feedback sessions, 116 women received usual care plus three computer-tailored feedback letters via the Internet, and 142 women received usual care or routine alcohol care from their nurse-midwives. The effect of the interventions on alcohol use was assessed at 3 or 6 months.

Results from this trial were promising overall because after 6 months and three feedback letters, the participants receiving computer-tailored feedback stopped using alcohol more often than did those receiving usual care (53/68 women or 78% versus 51/93 women or 55%, respectively; *p* = .04). Limitations to the generalizability of findings include the lack of statistical power and suboptimal implementation of the health counseling intervention; consequently, no effect size was published.

Ondersma et al. conducted a pilot study with 48 pregnant women at an urban prenatal care clinic who screened positive for alcohol risk—defined as scoring positive on the T-ACE (tolerance, annoyed, cut-down, eye-opener) questionnaire, as well as drinking weekly or more in the past month, or having four or more drinks at least once a month in the 12 months before becoming pregnant.^39^ Participants were randomized to either electronic screening and brief intervention (e-SBI) or a control session on infant nutrition. The e-SBI was a 20-minute interactive session based on motivational interviewing and self-determination theory, followed by three tailored mailings to participants. The follow-up assessment occurred in person after childbirth and before hospital discharge; it was blinded to treatment condition. Results for alcohol use and birth outcomes were of moderate size and favored the intervention (*OR* = 3.4, *p* = .19 and *OR* = 3.3, *p* = .09, respectively). Although this pilot study showed that the technology-delivered intervention was feasible and acceptable, it could not evaluate the separate contributions of the e-SBI and the mailings or provide an effect-size.

Montag et al. randomized 263 American Indian/Alaska Native women of childbearing age (including 29 pregnant women and 234 nonpregnant women) to a culturally targeted online SBIRT intervention (eCHECKUP TO GO) or treatment as usual.^40^ eCHECKUP TO GO is a web-based brief assessment and intervention. All participants completed a baseline survey that evaluated awareness of FASD, usual alcohol consumption, and demographic background among other factors. With little loss to follow-up (6%), the investigators found that risky drinking behavior (defined as three or more standard drinks per occasion and/or eight or more drinks per week) and risk of AEP were reduced in both the intervention and control groups. There was evidence of a time effect but no statistically significant treatment effect. Study limitations included self-selected volunteers (rather than women who met criteria such as being pregnant and drinking at certain levels) in addition to the usual concerns about self-report.

Ingersoll et al. conducted a pilot randomized trial of an Internet intervention to reduce the risk of AEP among 71 women drinking at a risky level (defined as four or more standard drinks per episode in the past 3 months) without effective or consistent contraception.^41^ The women were randomized either to a six-core automated, interactive, and tailored Internet intervention—the Contraception and Alcohol Risk Reduction Internet Intervention (CARRII) based on the CHOICES intervention—or to a static, untailored patient education website offering the same content as CARRII (e.g., information about AEP, FASD, and alcohol use among women). The investigators then assessed the intervention’s effect on AEP risk. Of the participants, 64 women completed 6-month follow-up. Women in both conditions reduced risky drinking by less than 20% at 6 months; however, those receiving CARRII demonstrated significant reductions in the proportion of unprotected sex from pretreatment to posttreatment (32%) and to 6-month follow-up (30%).

Wernette et al. conducted a two-group, randomized controlled trial of 50 pregnant women with an average of 13 weeks’ gestation attending an inner-city prenatal clinic.^42^ Inclusion criteria included pregnant women who endorsed (1) vaginal (or anal) sex without a condom at least once in the past 30 days, (2) unplanned pregnancy, and (3) current alcohol or drug use or at risk for the same because of a positive alcohol (T-ACE) or drug (Substance Use Risk Profile-Pregnancy) screen. The intervention group was given a computer-delivered, single-session brief motivational interview with booster session, both of which addressed substance use and risk of sexually transmitted infection. The attention control condition included answering questions related to television shows and providing subjective ratings. At 4-month follow-up, participants in the intervention arm had a significant reduction in any marijuana or alcohol use compared to the control arm (54% versus 16%, *p* = .015) but an insignificant reduction in vaginal sex without a condom. Potential limitations included reliance on self-report of risk behaviors, inclusion of only English-speaking participants, imbalance in randomization (31 in the intervention group, 19 in the control group), and unblinded research staff.

#### Use of other technology: 4D ultrasound

Jussila et al. conducted a novel, randomized controlled trial among pregnant women attending an obstetric outpatient clinic in Finland.^43^ Ninety women were referred to the clinic because of current or recent substance use—defined as self-reported or documented illicit substance use, abuse of prescription medication or alcohol within the past 3 years or during a previous pregnancy, and/or a score of 3 or higher on the Tolerance-Worry-Eye-Opener-Annoy-Cut-Down (TWEAK) alcohol screening tool. They were randomized either to a control group (*n* = 44) receiving treatment as usual or to an intervention group (*n* = 46) that included interactive use of 4D ultrasound visualization of the fetus at 24, 30, and 34 weeks and a pregnancy diary. The ultrasounds and diary were designed to enhance prenatal parental mentalization and maternal-fetal attachment. With an 89% retention rate overall, retention was significantly higher in the intervention group than in the control group (96% vs. 82%, *p* < .05). Although 74% of the intervention group participated in all three ultrasound sessions, only 59% participated in all scheduled obstetric sessions (compared to 83% of the control group, *p* < .02). Fetal drug exposure (as measured in meconium samples) and perinatal outcomes (i.e., rates of small for gestational age babies) were similar in both groups. This study did not focus on prenatal alcohol use, but included women at general risk of substance use.

In conclusion, the use of technology approaches for offering education and intervention appears to be a potentially promising approach to reducing AEP. However, most studies thus far have been small, reflecting their exploratory and innovative nature.

## Discussion and Conclusions

This narrative review summarizes the many well thought-out clinical trials and randomized clinical trials conducted in the last decade assessing four types of interventions to reduce preconception and prenatal alcohol use, the sole cause of FASD.

Although they were well intentioned, clinically appealing, and may help some individuals, case management and home visits did not appear to have strong empirical support. These efforts were challenging to implement even by the investigators and may be difficult to reproduce by others. Some studies were exploratory in nature and had small sample sizes and no comparison groups, whereas larger efforts did not result in definitive advantages for this intensive approach.^20^–^23^

Interventions aimed at women prior to conception, which generally were based on the Project CHOICES approach,^24^ had similar outcomes. In these studies, the reduction in AEP risk was largely realized by improved contraception in women of childbearing age who were sexually active and drank alcohol while not pregnant.^25^,^27^–^30^ Although the Project CHOICES-based efforts used well-defined strategies and designs, it is unknown whether these women refrained from alcohol use when they became pregnant in the future.^44^

Two studies of motivational interviewing to reduce prenatal alcohol use published in the past decade did not demonstrate the efficacy of the intervention.^31^,^32^ Both studies were small, including fewer than 200 pregnant women combined. Another limitation was low baseline levels of alcohol use in the study sample, which allowed for little room for improvement.

Finally, several studies evaluating the impact of technology were reviewed. Most had small sample sizes as they were exploratory in nature. However, their contributions include preliminary evaluations of techniques such as text messages, telephone contact, as well as computer-based screening and motivational interviewing that may inform future research and clinical efforts, particularly as the COVID-19 pandemic has impacted how treatment may be delivered in the future.^33^–^42^ A recent meta-analysis of six of the 10 studies included in this review supports the potential of digital interventions.^45^

## Future Directions

The findings described in this review indicate the need for research to address the limitations of the evidence to date. As there may be other potential obstacles to progress, prevention of AEP and the incidence of FASD will require a multipronged approach. First, FASD is one of the most complex developmental disabilities, and although prenatal alcohol exposure is the proximal teratogen, it rarely occurs in isolation.^46^ The etiology of FASD also includes a range of lifestyle, sociodemographic, maternal, social, gestational, and genetic factors that need to be considered.^3^ Future studies may include systematic collection of prespecified variables selected by expert panels to ensure consistency and comparability of studies.

Second, many of the past studies have had a narrow focus that did not take into consideration the complexity of modern childbearing. For example, punitive laws associated with prenatal substance use may discourage patient disclosure and hence limit opportunities for clinical intervention.^47^,^48^ There is continued controversy in some nations over whether the law should intervene when a pregnant woman’s actions may risk serious and preventable fetal injury.^49^,^50^ The full impact of punitive laws on alcohol and substance use by pregnant women remains to be fully evaluated in future research; however, it is notable that the United States does not criminalize or punish women with other health conditions (e.g., diabetes, epilepsy, obesity) that can affect their pregnancy or their children.^47^

Third, although it is desirable to base clinical practices on sound research, the demand for evidence-based practice may have unintended consequences in clinical work or the ability to offer guidance to patients and practitioners alike as long as a definitive approach remains elusive.^5^,^6^,^51^ For example, some have concluded that the absence of a safe drinking limit during pregnancy means that some alcohol use during pregnancy is acceptable.^52^,^53^ Others have recognized that the lack of evidence is linked with a lack of dedicated funding and services, and consequently a lack of policy formulation and strategic direction in the United Kingdom^54^ and Africa;^55^ other types of gaps have been identified in Canada.^56^,^57^ Thus, the impact of research is potentially far-reaching. As such, it may be time to understand the limits of conventional research designed to optimize efficacy, and to consider the potential contribution of more pragmatic approaches.^58^

Finally, two problematic secular trends have emerged in recent years: the increase in alcohol use among women during the COVID-19 pandemic^59^,^60^ and a growing mistrust of science and professional advice that has historical roots.^61^,^62^ For example, reliance on information disseminated by organizations funded by the alcohol industry, including mandatory pregnancy health warning labels on alcohol, may reduce the willingness of pregnant women to stop drinking alcohol.^63^,^64^ Thus, too many people remain unconvinced that prenatal alcohol use is unsafe, or they do not understand the more nuanced rationale for abstinence. Future research may need to take into account the social contexts of problematic or risky behaviors so that interventions can be effectively implemented.

With 14% of pregnant women reporting alcohol use between 2018 and 2020, the Healthy People 2030 goal of 92% prenatal abstinence is a stretch. To reach that goal and optimize pregnancy outcomes, the next steps may require bold and creative thinking that goes beyond the well-studied preconception approaches, such as evaluation of those using video and other technologies that are consonant with patients and treatment professionals alike. Collection of consistent data sets to advance knowledge also may be helpful to reach the goals.^3^ The costs associated with FASD are high and may be even higher than currently realized;^65^–^67^ therefore, additional efforts to identify effective ways to reduce the risk of AEP are clearly warranted.

## Figures and Tables

**Table 1:** Literature Search Tracking Summary

Search #	Date of Search	Database	Search Terms	Number of Hits	Excluded

1	November 2021	PubMed	(“alcohol drinking”[MeSH Major Topic] OR prenatal alcohol[Title/Abstract]) AND (“Pregnancy”[MeSH Terms] OR “Prenatal Care”[MeSH Terms]) AND (“clinical trial”[Publication Type] OR “clinical trial, phase I”[Publication Type] OR “clinical trial, phase II”[Publication Type] OR “clinical trial, phase III”[Publication Type] OR “clinical trial, phase IV”[Publication Type] OR “controlled clinical trial”[Publication Type] OR “pragmatic clinical trial”[Publication Type] OR “randomized controlled trial”[Publication Type])	94	20 duplicates 20 no abstract/content 12 secondary analysis 6 treatment of children with FASD 6 PAE effects 3 protocol only 3 maternal factors 1 prevalence 1 biomarker 1 cross-sectional 1 postpartum 1 universal
		Ovid MEDLINE	(prenatal* adj5 (alcohol or ethanol)).tw. limited to (clinical trial, phase I or clinical trial, phase II or clinical trial, phase III or clinical trial, phase IV or clinical trial or controlled clinical trial or pragmatic clinical trial or randomized controlled trial)		
		Clinical Key	“prenatal alcohol” filtered by Clinical Trials		
		WHO ICTRP and ClinicalTrials.gov	“prenatal alcohol”		
			Search limited to articles published in English over the last 10 years.		**Yield: 19 included**

2	May 2022	PubMed	prevention AND fetal alcohol spectrum disorders	19	13 duplicates of Search 1 4 outside review scope (3 on FASD effects, 1 on beliefs)
			Search limited to articles on randomized clinical trials and clinical trials published in English over the past 10 years.		**Yield: 2 included**

3	May 2022	PubMed	PCAP (parent child assistance program)	125	119 acronyms for other medical entities 4 secondary analyses 1 duplicate 1 qualitative study
			Search limited to articles published in English over the past 10 years.		**Yield: 0 included**

*Note:* FASD, fetal alcohol spectrum disorders; MeSH, Medical Subject Headings; PAE, prenatal alcohol exposure; PCAP, parent-child assistance program; WHO ICTRP, World Health Organization International Clinical Trials Registry Platform.

**Table 2:** Study Summaries

Case Management		
Author	Design	Main Findings/Comments
May et al., 2013^20^	Prospective intervention study of women at high risk of drinking (*n* = 41); 88% pregnant at enrollment. CM with 6-, 12-, and 18-month follow-up.	Although it is difficult to achieve enduring change in drinking in this group, pregnant women did drink less at 6- and 18-month follow-up.
De Vries et al., 2016^21^	Prospective intervention study of pregnant South African women (*n* = 67). Complete data available for 50 of 67 women; no comparison group. CM with drinking measured at baseline and at 6, 12, and 18 months.	Mean reductions in drinking were seen at 6 months, but higher levels were observed at 12 and 18 months over baseline.
Rotheram-Borus et al., 2019^22^	Longitudinal cluster randomized trial of pregnant mothers (*n* = 1,236) from 24 different South African neighborhoods. Severely impoverished sample (e.g., 53% with running water). Standard clinical care vs. CM (Philani Program) with home visits/paraprofessional coaching on drinking from pregnancy to 5 years post-birth.	Women in the Philani Program drank less postnatally, but all women gradually returned to pre-pregnancy rates of drinking.
Catherine et al., 2020^23^	Analysis of prenatal secondary outcomes in an ongoing RCT of pregnant women (*n* = 739). NFP with CM + existing services vs. existing services only. Analyses were intention-to-treat and mixed-effect models for longitudinal and clustered data to estimate intervention effects.	NFP had no effect on reducing rates of prenatal use of cigarettes or alcohol, but did lead to reduced prenatal use of cannabis.

Preconception Trials to Prevent AEP		
Author	Design	Main Findings/Comments
Rendall-Mkosi et al., 2013^25^	RCT of MI in women at risk for AEP (*n* = 165). Five-session MI intervention, timed and structured evaluations preintervention and 3 and 12 months afterwards. Outcome was AEP at 12 months, modeled on Project CHOICES. Three conditions: MI, life skills, and control. Life skills stopped after 30 days due to poor adherence.	The MI group was more than twice as likely as the control group to lower their risk for AEP at 12 months (*OR* = 2.64); intention-to-treat analysis reduced the odds ratio to 2.19. Reduction in AEP risk was due mainly to the use of contraception rather than reduced drinking. There was discussion that, compared with other health professionals, medical doctors have greater success in effecting behavior changes.
Ingersoll et al., 2013^26^	RCT to test a one-session MI to reduce AEP in community women (*n* = 217), to either MI + assessment feedback (EARLY), informational video vs. informational brochure.	All interventions associated with drinks per drinking day, ineffective contraception rate, and AEP risk at 3 and 6 months. One-session EARLY intervention had less powerful effects than multiple sessions on AEP.
Sobell et al., 2017^28^	RCT of women at risk for AEP (*n* = 354); 44% minorities. MI is based on CHOICES vs. information only.	No significant difference was seen between the interventions. Comment: “The most effective AEP prevention strategy is to simply communicate to those at risk that they could become pregnant.”
Velasquez et al., 2017^29^	Women ages 18–44, not pregnant, not sterile, drinking > 3 drinks per drinking day or > 7 drinks per week, sexually active, not using effective contraception. RCT with two intervention groups: CHOICES Plus (*n* = 131) vs. brief advice (*n* = 130) in 12 primary care clinics in large Texas public health system.	Primary outcomes were reduced risk for AEP and TEP through 9-month follow-up. Intention-to-treat analysis across 9 months and CHOICES Plus significantly reduced risk of AEP and TEP.
Hanson et al., 2015^30^	American Indian women, not pregnant, enrolled in the Ogala Sioux Tribe CHOICES program (*n* = 193) and at risk for AEP.	51% completed 3- and 6-month follow-up. Risk for AEP reduced by improved contraception rather than reduced binge drinking.

Motivational Interviewing/SBIRT		
Author	Design	Main Findings/Comments
Osterman & Dyehouse, 2012^31^	RCT of MI compared to comparison group (*n* = 67); 56 pregnant women completed all study procedures. Self-determination theory applied to increase understanding of the mechanisms of MI. Two group pretest and posttest study of convenience sample. Three basic psychological needs: autonomy, competence, relatedness.	Structural equation modeling determined the direct, indirect, and total effects on the MI intervention on outcomes. Unexpected findings were that MI intervention had no significant effects in decreasing prenatal drinking behaviors. Intervention theory-based specific and nonspecific factors drive effective nursing interventions.
Osterman et al., 2014^32^	RCT of pregnant women (*n* = 122) from three prenatal clinics in the Midwest, who drank any amount of alcohol in the previous year; 64% African American. Intervention or no-intervention comparison group, pretest/posttest design.	MI was ineffective in decreasing drinking.

Use of Technology		
Author	Design	Main Findings/Comments
Evans et al., 2012^34^	RCT of pregnant women (*n* = 123) first presenting for care at the Fairfax County, Virginia, Health Department. All with UC; Text4baby vs. UC alone.	There was a 73% retention rate at 28 weeks’ gestation. Attitudes toward alcohol consumption improved from baseline to follow-up (*OR* = 2.57, 95% *CI* [1.13–11.24], *p* = .03).
Evans et al., 2014^35^	RCT of military women (*n* = 943); pregnant < 14 weeks, first presenting for prenatal care. All with UC; Text4 baby + UC vs. UC alone.	49% completed a 4-week follow-up survey. Beliefs about avoiding alcohol and attending health care appointments improved, but there were no changes in self-reported behavior.
Farrell-Carnahan et al., 2013^36^	Uncontrolled prospective pilot study of non–treatment-seeking community women at risk of AEP (*n* = 46). One-session MI-based AEP intervention via telephone with 3- and 6-month follow-up.	Reduction in drinking and improved use of effective contraception. Telephone may not be as potent as longer, face-to-face contact.
Wilton et al., 2013^37^	Women who screened positive for risky drinking and not using effective contraception (*n* = 131). RCT of intervention given via telephone vs. in person, followed for 6 months.	Women who were Black and unemployed were more likely to be randomized to in-person contact. Intervention modality was a significant predictor of outcomes. Overall, both groups had small but significant reductions in alcohol use and larger increases in use of effective contraception. 73% completion rate.
van der Wulp et al., 2014^38^	Dutch midwifery practices (*n* = 60) randomized to one of three conditions: health counseling, CT, or UC/routine alcohol care. CT patients received UC + three CT feedback letters. Health counseling was manual-driven with three feedback sessions. Follow-up at 3 and 6 months.	The CT group stopped using alcohol more often than the UC group. Health counseling was not given consistently, and reductions in alcohol use did not differ between CT and UC.
Ondersma et al. 2015^39^	Pregnant women (*n* =48) who screened positive for alcohol risk at an urban prenatal care clinic. RCT of e-SBI + three tailored mailings vs. control session on infant nutrition. Primary outcome was 90-day prevalence of abstinence.	Nonsignificant findings for underpowered pilot. e-SBI + tailored mailings were well received. Medium-sized intervention effects on 90-day period prevalence estimate.
Montag et al., 2015^40^	Non–treatment-seeking sample of American Indian/Alaska Native women of childbearing age (*n* = 263), randomized to online SBIRT or treatment as usual after assessment at 1-, 3-, and 6-month follow-up. One-third of sample was at risk for AEP.	Both the SBIRT and treatment as usual groups showed reduced alcohol use after enrollment.
Ingersoll et al., 2018^41^	Women ages 18–44, fertile, with Internet/telephone access, risky drinking, and at risk for unintended pregnancy for past 3 months (*n* = 71). RCT of 6-core automated, interactive, and tailored Contraception and Alcohol Risk Reduction Internet Intervention to static patient education.	64 of 71 women completed at least one part of 6-month assessment. The rate of unprotected sex significantly reduced from pretreatment to posttreatment and at 6-month follow-up. Reductions in risky drinking were seen from pretreatment to posttreatment, but not at 6 months. Rate of AEP pretreatment (67%) reduced to 32% posttreatment and 30% at 6 months. Using a combined pregnancy outcome variable, neither an intent-to-treat analysis nor a group by treatment analysis were statistically significant.
Wernette et al., 2018^42^	RCT of pregnant women (*n* = 50) at a prenatal clinic in a large inner-city hospital. Computer-delivered, single session brief motivational intervention + booster session addressing substance use disorder and significant risk for sexually transmitted infection vs. control group.	Intervention was acceptable to participants. The intervention group had a 54% reduction in marijuana and alcohol use compared to the control group (16%) (*p* = .0015). There was a nonsignificant reduction in vaginal sex without a condom.
Jussila et al., 2020^43^	RCT of pregnant women referred to a hospital OB outpatient clinic due to recent or current substance use (*n* = 90), all treatment as usual. Intervention groups: three interactive US, pregnancy diary, and three prenatal infant consultations. 4D US thought to enhance parental mentalization and prenatal attachment.	89% retention rate, intervention group > control. 74% attended all three US sessions. Women in the intervention group attended fewer OB sessions than those in the control group (59% vs. 83%, *p* = .02). Fetal drug exposure and perinatal outcomes were similar in both groups.

*Note:* 4D, four-dimensional; AEP, alcohol-exposed pregnancy; *CI*, confidence interval; CM, case management; CT, computer tailoring; EARLY, MI + assessment feedback; e-SBI, electronic screening and brief intervention; MI, motivational interviewing; NFP, nurse-family partnership; OB, obstetrics; *OR*, odds ratio; RCT, randomized controlled trial; SBI, screening and brief intervention; SBIRT, screening, brief intervention, and referral to treatment; TEP, tobacco-exposed pregnancy; UC, usual care; US, ultrasound.
